# Gathering and Exploring Scientific Knowledge in Pharmacovigilance

**DOI:** 10.1371/journal.pone.0083016

**Published:** 2013-12-11

**Authors:** Pedro Lopes, Tiago Nunes, David Campos, Laura Ines Furlong, Anna Bauer-Mehren, Ferran Sanz, Maria Carmen Carrascosa, Jordi Mestres, Jan Kors, Bharat Singh, Erik van Mulligen, Johan Van der Lei, Gayo Diallo, Paul Avillach, Ernst Ahlberg, Scott Boyer, Carlos Diaz, José Luís Oliveira

**Affiliations:** 1 DETI/IEETA, University of Aveiro, Aveiro, Portugal; 2 Research Programme on Biomedical Informatics (GRIB), IMIM Hospital del Mar Research Institute and Universitat Pompeu Fabra, Barcelona, Spain; 3 Erasmus University Medical Center, Rotterdam, The Netherlands; 4 LESIM-ISPED, Université de Bordeaux, Bordeaux, France; 5 LERTIM, EA 3283, Faculté de Médecine, Université de Aix-Marseille, Marseille, France; 6 AstraZeneca, Molndal, Sweden; 7 Synapse Research Management Partners, Barcelona, Spain; Royal College of Surgeons, Ireland

## Abstract

Pharmacovigilance plays a key role in the healthcare domain through the assessment, monitoring and discovery of interactions amongst drugs and their effects in the human organism. However, technological advances in this field have been slowing down over the last decade due to miscellaneous legal, ethical and methodological constraints. Pharmaceutical companies started to realize that collaborative and integrative approaches boost current drug research and development processes. Hence, new strategies are required to connect researchers, datasets, biomedical knowledge and analysis algorithms, allowing them to fully exploit the true value behind state-of-the-art pharmacovigilance efforts. This manuscript introduces a new platform directed towards pharmacovigilance knowledge providers. This system, based on a service-oriented architecture, adopts a plugin-based approach to solve fundamental pharmacovigilance software challenges. With the wealth of collected clinical and pharmaceutical data, it is now possible to connect knowledge providers’ analysis and exploration algorithms with real data. As a result, new strategies allow a faster identification of high-risk interactions between marketed drugs and adverse events, and enable the automated uncovering of scientific evidence behind them. With this architecture, the pharmacovigilance field has a new platform to coordinate large-scale drug evaluation efforts in a unique ecosystem, publicly available at http://bioinformatics.ua.pt/euadr/.

## Introduction

Pharmacovigilance plays an essential role in the post-market analysis of newly developed drugs [1, 2]. Pharmaceutical companies' competition along with rigorous regulatory evaluation procedures empowers a complex research and development process before launching a new drug into the market. Notwithstanding, drug safety continues to be a relevant concern for healthcare involving worldwide policy stakeholders, from regulatory health authorities to specialised law firms. Post-market pharmacovigilance complements the traditional pre-market austere drug approval process, where the European Medicines Agency (EMA) [3] and the US Food and Drug Administration (FDA) [4] establish guidelines for new medicine approval, requiring intense testing and trials [5]. Along with these recommendations, pharmaceutical companies must also define thorough risk management plans for post-market drug stages [6, 7].

Pharmacovigilance research is based on the analysis of "signals". The World Health Organization (WHO) defines signals as undisclosed assertions on direct relationships between adverse events – effects on the human organism - and a drug [8]. To generate comprehensive signal datasets, clinicians and researchers use spontaneous reporting systems (SRS). Electronic SRSs are already in place throughout some European countries and the USA. Likewise, other solutions, such as general practitioners’ databases analysis, post market studies or prescription monitoring, among others, are being thoroughly explored. Nevertheless, the majority of data is not publicly available for researchers, which, jointly with other barriers, severely limits signal detection [9, 10].

Although drug companies are required to track and manage adverse events reported by clinicians, lawyers or patients, the detection process relies mostly on the physician's ability to recognise a given trait as a drug adverse event. In addition to this underreporting, results are also biased due to selective reporting (reporting only certain drugs or conditions), placing the threshold of reported ADRs between 1-10% [11-13].

Whereas the problem for collecting and filtering ADR data from multiple distributed nodes has already been studied in the past [14], researchers continue to pursuit the best strategies to delve into the wealth of collected data in conjunction with other post drug administration inputs. With data and text-mining techniques scavenging millions of electronic medical records, pharmacovigilance researchers are now faced with the problem of delivering knowledge-oriented tools and services that exploit the scope of collected data. Ultimately, the adequate exploration of these data will pave the way for improved drug evaluations, critical for pharmaceutical companies, regulatory entities and researchers [15].

And herein lies the grand problem for contemporary pharmacovigilance: how to enable any researcher to assess and explore the wealth of collected data across a variety of algorithms and tools? In summary, researchers need new automated strategies to mechanistically understand the scientific evidence behind specific drug and adverse event interactions, through the processing of data mined from millions of electronic medical records and analysed independently by multiple algorithms. To overcome this problem, six key challenges arise for researchers and developers.

• **Scalability**. Controlling a flexible amount of algorithms, each providing independent access to knowledge, with its independent set of features and offering access to closed functionalities.
• **Interoperability**. The integration of multiple knowledge providers requires that a solution akin to a "common language" must be setup so that the various tools and algorithms can interact with each other and with a central software choreographer.
• **Management**. This brings two challengers: (1) how to store and make the collected data available to all researchers, and (2) how to organise and coordinate the set of available knowledge providers.
• **Reproducibility**. The replication of all research steps, including data and used knowledge providers must be available for other researchers and for further auditing.
• **Accessibility**. All the data and features must be presented in a unified workspace, publicly available to all interested stakeholders.
• **Security**. At last, interactions between knowledge providers, implemented software and researchers must be established through secure channels.

The strategy introduced in this manuscript, and its underlying architecture, implementation and prototype, successfully covers the aforementioned challenges, introducing a pioneering solution to deliver pharmacovigilance studies to researchers worldwide.

## Materials and Methods

### Background

Large-scale projects such as Research on Adverse Drug Events and Reports (RADAR) [16], Observational Medical Outcomes Partnership (OMOP) , Mini-Sentinel [17] or Exploring and Understanding Adverse Drug Reactions by Integrative Mining of Clinical Records and Biomedical Knowledge (EU-ADR) [18], among others, are pushing forward innovative strategies to improve active pharmacovigilance scenarios. 

The RADAR project adopts a strategy where a highly specialized team reviews incoming drug adverse reports. Despite generating invaluable curated results, this approach implies a large waste of intensive manual labour. In opposition, OMOP, Mini-Sentinel and EU-ADR adopt more automated strategies. 

OMOP and EU-ADR share a common setup, where data from partners are automatically collected, mined and analysed. Partners’ data are translated to a common data model (CDM), anonymized, summarized and imported into a central integrative data repository for further statistical processing.

 OMOP is applied to two distinct surveillance scenarios, tackling the identification of well-known drug associations and the identification of previously unknown signals [19]. This identification is validated through the application of multiple analytical procedures over a broad number of summarized patient records. In fact, OMOP’s initial stage finished with an assessment of the best methods to identify pharmaceutical risk in healthcare data [20]. Selected algorithms are now being applied in the project’s second stage.

EU-ADR distributed pipeline, discussed in detail in the following section, is very similar to OMOP’s. The major difference resides on the partners’ data. Whereas in OMOP most data stems from private contractors in the United States of America, in EU-ADR, data are obtained from European nationwide registries. Regarding the statistical analysis, EU-ADR’s core longitudinal observation algorithms are LGPS and LEOPARD [21].

Despite featuring a distributed architecture, Mini-Sentinel is very different from the setup used in OMOP and EU-ADR. Whereas in the latter projects data are summarized and submitted for statistical analysis, in Mini-Sentinel data queries go through a complex network [22]. Like similar projects, partners in the Mini-Sentinel program translate their dataset to the project’s CDM. However, in Mini-Sentinel, data never leaves the original institution [23]. This addresses FDA’s concerns regarding security and privacy.

With Mini-Sentinel’s strategy, data queries are “executable programs” that are sent to partners for in-premises execution [24]. Once queries are received, partners can analyse requested data, execute them and validate the results before reporting to the query authors. Mini-Sentinel’s project coordination then assembles generated results and transfers summary data to the query authors. In spite of being more secure, this strategy can lead to delays in query answering, specially considering the project’s 7-day average response time.

With most of these projects in their infancy, a correct assessment of their results is a long-term task. Nevertheless, comparison frameworks are being put in place to better evaluate and compare the qualitative results of each project [25].

### The EU-ADR Project

**Figure 1:**
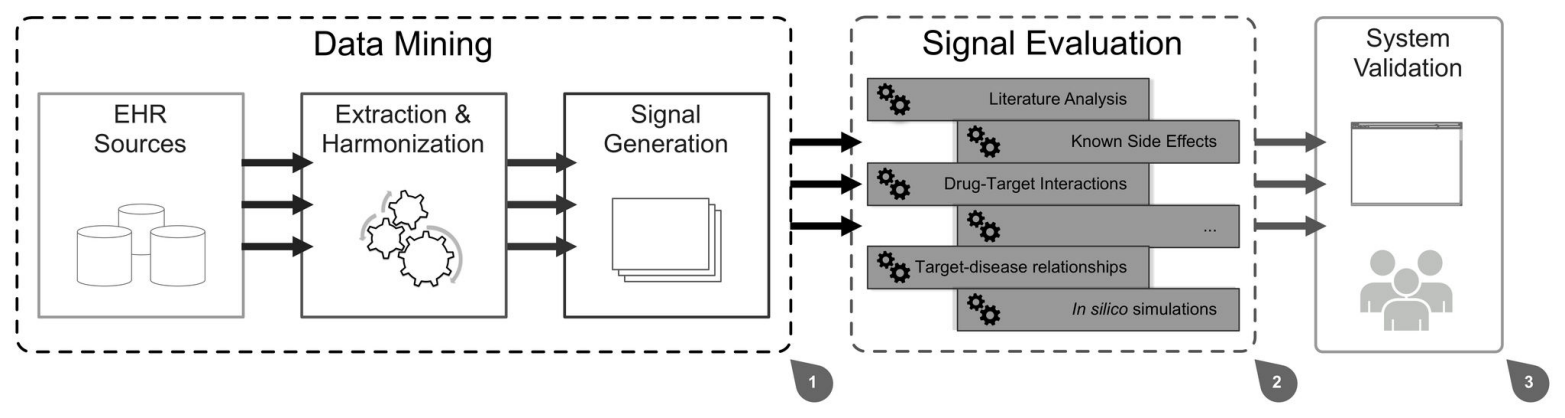
EU-ADR initiative data flow. 1) Data extracted from electronic health record (EHR) resources are semantically harmonized for data mining, generating a raw drug-event pair list. 2) The signal substantiation process analyses the submitted data, re-ranking the signal list, based on multiple algorithms. 3) Users trigger data analysis and exploration to validate the system operability.

The foundation for EU-ADR's strategy relies on in-depth semantic data mining of electronic health records from several European countries. This process generates filtered data that can be easily substantiated through distributed computational tools [26] – Figure 1.

Project partners provide demographics, drug use and clinical data for over 20 million patients from several European countries. These data include clinical history, drug prescriptions, vaccinations, or lab test results [27, 28]. From a pharmacovigilance perspective and in a European or worldwide scale, mining the amount and type of data collected in these databases is of tremendous importance for an improved post-marketing drug evaluation [29].

Data mining techniques are used to extract the most relevant information from these data sources [30, 31], taking in account the privacy and ethical concerns regarding the collected data [32]. Next, data are harmonized into a unified dataset containing the list of drug-event pairs identified in the mined records [33]. This initial detection process generates a raw signal list, as the signal detection techniques highlight all possible relationships discovered in the wealth of collected data [34] – Figure 1-1.

**Figure 2:**
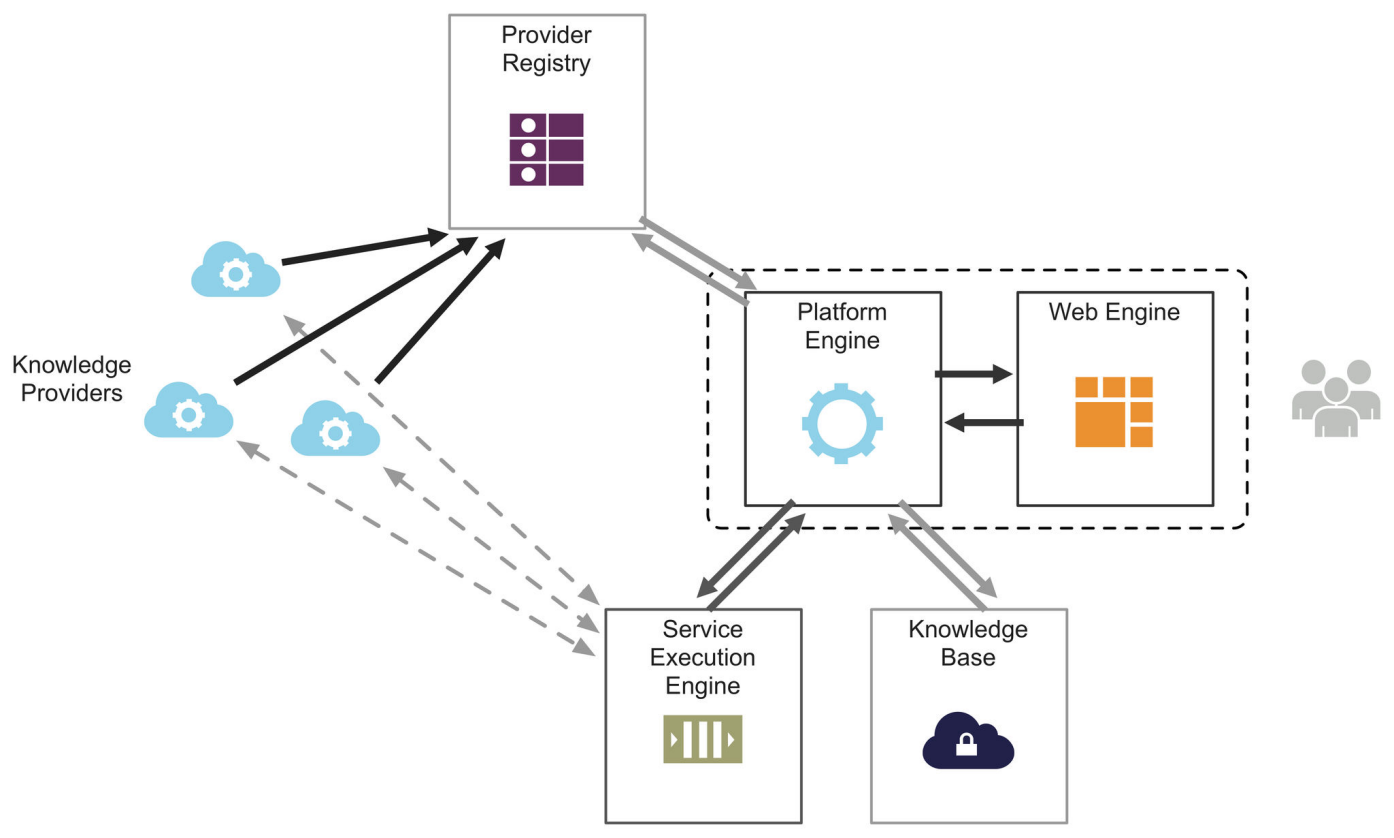
General architecture for the distributed pharmacovigilance platform.

As mentioned, the creation of these rich signal datasets is a well-established task. However, the actual data analysis and exploration tasks are still missing. Each signal in the raw list provided by the data mining tools must be substantiated for adequate validation. That is, the signal must be analysed by multiple algorithms to identify its real risk, and, if it exists, to provide a scientific explanation behind the cause, the drug, and the effect, a specific adverse reaction. This step, highlighted in Figure 1-2, differentiates the EU-ADR project pipeline from other related projects.

The signal substantiation algorithms can range from simple literature analysis, to drug target interaction matching. This is the key pharmacovigilance challenge to the EU-ADR project: how to design an architecture that can leverage on the data acquired from millions of electronic health records by enhancing its automated evaluation through any number and kind of distributed algorithms? 

**Figure 3:**
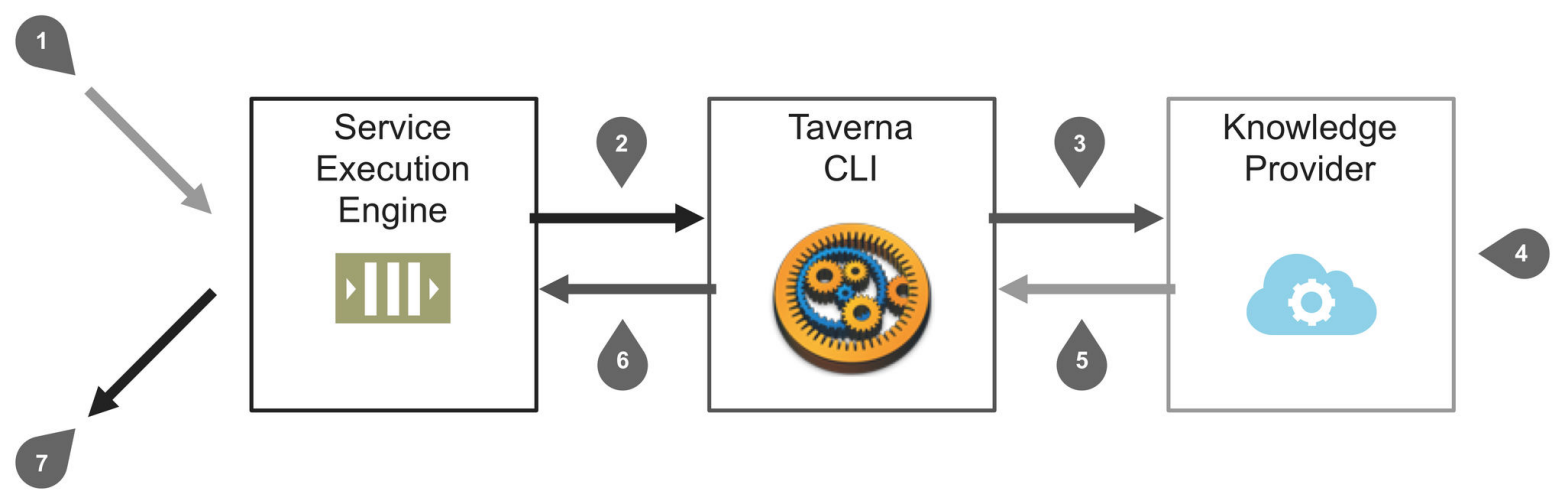
From user input to system output the platform engine controls the execution of workflows as follows: 1) One or more knowledge provider algorithms are selected to evaluate researcher-submitted datasets. The platform engine sends the request to the service execution engine. 2) An XML file with the input data (obeying the platform’s interoperability standard) is generated and its path provided to the service execution engine, along with the path for the workflows associated with each knowledge provider algorithm. The workflow execution is then triggered by a system call. 3) The Taverna command line tool loads the knowledge provider’s workflow, starting the processing tasks. 4) The knowledge provider execution proceeds internally, executing the miscellaneous workflow tasks. 5) The workflow delivers an XML data file (obeying the platform’s interoperability standard) with the algorithm output. 6) The service execution engine loads the XML output file and transfers the results to the platform engine. 7) The engine stores the data in the knowledge base and makes it promptly available for delivery in the web workspace.

At last, results for these algorithms must be easily available for researchers [35] – Figure 1-3. The data flow ends at the researchers’ workspace, where they can validate the system, explore the resulting scientific evidence and, if required, proceed to take the necessary steps to prevent new occurrences for the high-risk drug event interactions.

### A Distributed Pharmacovigilance Platform

The architecture of a distributed platform in the context of pharmacovigilance must tackle the six mentioned challenges - scalability, interoperability, management, reproducibility, accessibility and security. The proposed service-oriented architecture is shown in Figure 2 and its components described in Table 1.

**Table 1:** Architecture component descriptions, implementation and operability purpose.

Component	Operability	Implementation	Description
Service execution engine	External	Java Taverna	With each knowledge provider delivering service-based access to its algorithm, the service execution engine is responsible for performing the service calls with the input data read from the knowledge base and retrieving the output data towards the platform.
Knowledge base	Internal	Cloud-based	The knowledge base stores all relevant data from the integrated and imported pharmacovigilance datasets. Data are stored in a cloud-based environment, moving the inherent complexities associated with secure data storage to en efficient cloud provider.
Provider registry	Internal	Java	The provider registry acts as the main knowledge provider controller. This is where new knowledge providers must register their interfaces and endpoints so that they can be made available for future use.
Knowledge Providers	External	Independent XML-based standard	The knowledge providers deliver independent access to various pharmacovigilance data analysis and exploration algorithms. Access to knowledge providers is service-based.
Platform engine	Internal	Java	The platform engine is the architecture core component, where all the tasks are executed and the interactions controlled.
Web engine	Internal	Google Web Toolkit	The web engine powers the distributed platform user interactions through an innovative web-based workspace.

This architecture is built on top of multiple interactions, exploiting the components’ dynamics. Results from the semantic harmonization of mined records, the raw signal list, are securely stored on the knowledge base, being easily accessible to all the other components. Once the users select the knowledge providers from the provider registry, the data are transmitted to the service execution engine, which then contacts each of the knowledge providers for service execution. The outputs of the analysis algorithms are next stored in the platform’s knowledge base, and delivered to the researchers through the web application engine. All these interactions are controlled and securely mediated by the application engine.

#### Scalability & Interoperability

The provider registry and the service execution engine ensure scalability and interoperability. To completely remove any interdependence amongst knowledge providers, a common data description and exchange language was created. Additionally, to foster the seamless integration of knowledge providers, data input and output formats must be compatible.

A new XML schema (XSD) was designed to accomplish this. The flexibility of XSD enables the validation of both content and structure, preventing erroneous data transfers and reinforcing the overall platform robustness. The schema structure is divided in three main sections, each focusing on one operability perspective.

• **Monitoring**. Real-world use of knowledge providers can result in an assorted amount of errors: general communication errors, such as failure to connect to a database, or domain-specific errors, such as invalid data. Therefore, the created schema covers the domain-specific errors with a set of “status codes” for each of the possible conditions. For example, status code with the value “41” identifies an internal service problem regarding the database connection.
• **Scoring**. Each of the signals in the ranked list has a score that determines its relevant risk within the dataset. When the data are being assessed by the knowledge providers, the scoring attributes will provide each evaluated signal with a numeric value, between 0 (zero) and 1 (one), measuring the relative relevance and impact according to the scientific evidence found to explain the interactions of a given drug-event pair.
• **Annotation**. When a scientific explanation is found for a given set of drug-event pairs, the output is annotated with reliable evidence for the interaction, providing researchers with valuable knowledge and allowing them to evaluate the signal, share the results and reproduce their research in the future. These annotations appear in the form of connections to relevant resources, such as literature (PubMed links), proteins (UniProt links), chemical compounds (SMILE codes) or pathways (Reactome links), among others.

The schema is available online (http://bioinformatics.ua.pt/euadr/euadr_types.xsd), enabling anyone to create and add new algorithms to this plugin-based distributed platform, thus becoming one of the project's knowledge providers.

#### Knowledge Providers

Interoperability amongst various knowledge providers required the design of a strategy to explore the true value of the created data exchange standard. Whilst the schema is an essential component of the distributed platform interoperability features, it is useless by itself: the execution of knowledge providers’ algorithms must be intermediated by a distinct component, the service execution engine.

Another drawback regarding the implementation of knowledge providers relates to their internal algorithms. Whereas in some cases the algorithms are relatively straightforward, in the majority of scenarios the algorithms require multiple service-service interactions and data processing tasks.

This added another complexity layer to our architecture: the knowledge providers required heterogeneous interactions within their algorithms, a challenge that could not be tackled at the distributed platform level. Hence, the use of scientific workflows arises as a solution [36]. A crucial workflow requirement is that the inputs of each activity must match the precedent activity outputs to maintain consistency, a feature already accomplished with the platform interoperability standard. Dealing with workflow execution operations requires the implementation of workflow management applications, whose goal is to abstract the programming side of the application, assisting in the creation of workflows without writing a single line of code [37].

Taverna emerged as the *de facto* standard for desktop-based workflow management in the life sciences [38]. Taverna’s success is due to its flexibility, which allows researchers to create complex workflow-based algorithms just by dragging and dropping boxes in its workbench. Alternatives to Taverna, such as Galaxy [39] or BioFlow [40], are focused on providing workflow management functionality in a web-based interface. However, this was not a requirement for our scenario and, at the time of development, these tools do not offer an API as advanced as Taverna’s.

With Taverna in place, the provider registry collects metadata for Taverna workflows, and contains algorithms that can be downloaded for local use or executed online in the distributed platform. In addition to maintaining a list of available workflows, Taverna’s integration also required the implementation of a service execution engine. This solution allows the combination of comprehensive data analysis and exploration algorithms within the distributed platform. In summary, we need to feed the workflows with XML input data, execute them and extract the resulting data from the XML output. Figure 3 illustrates the steps required to execute knowledge providers’ workflows.

The service execution engine is a Java tool built to execute Taverna’s command line interface with custom input arguments. These parameterized system calls run in their own independent OS process, increasing the overall platform performance and scalability. Workflow executions are also a background non-blocking asynchronous process. For researchers, this means that they can use all the application features whilst the workflows are being executed in the background.

#### Knowledge Management

The adequate management of scientific data is critical to the success of the proposed distributed pharmacovigilance platform. Not only we need to consider how to make all relevant knowledge accessible at all times, we also need to implement adequate data sharing features: data must be exchanged between knowledge providers and collaboration is one of the underlying premises for research reproducibility.

The knowledge base is stored on a cloud environment [41]. This means that while the underlying data storage layer is distributed through multiple independent data nodes, the access is unified and centralized through a single access point. Common data storage issues such as persistence, security and access are controlled by the cloud-based layer, leaving the relevant data handling tasks to the platform engine [42]. 

In the EU-ADR project context, five key datasets are stored, detailed next.

• **Drugs**. Dataset containing the complete list of ATC codes and respective drug names.
• **Adverse events**. Dataset listing the adverse events mined from the project's pharmacovigilance data.
• **Imported data**. Researcher-submitted datasets containing statistical data regarding specific drug-event mapping conditions.
• **Results**. Datasets with the results from the knowledge providers' algorithms.
• **Users**. Dataset containing the user details and sharing/collaboration preferences.

Collaboration features are implemented according to two distinct methods: project-based and *ad hoc* sharing. With the project-based collaboration option, new *projects* with any number of users can be configured. This allows a broad number of users to manage a topic-specific dataset, fostering a deeper collaboration amongst researchers through the sharing of submitted data and obtained results.

*Ad hoc* collaboration is a user-specific approach. Users can share their datasets and results to any other user in the platform through their registration email. This is a more granular approach, where the users can define what data to share and what their collaborators can view or change.

#### Accessibility

**Figure 4:**
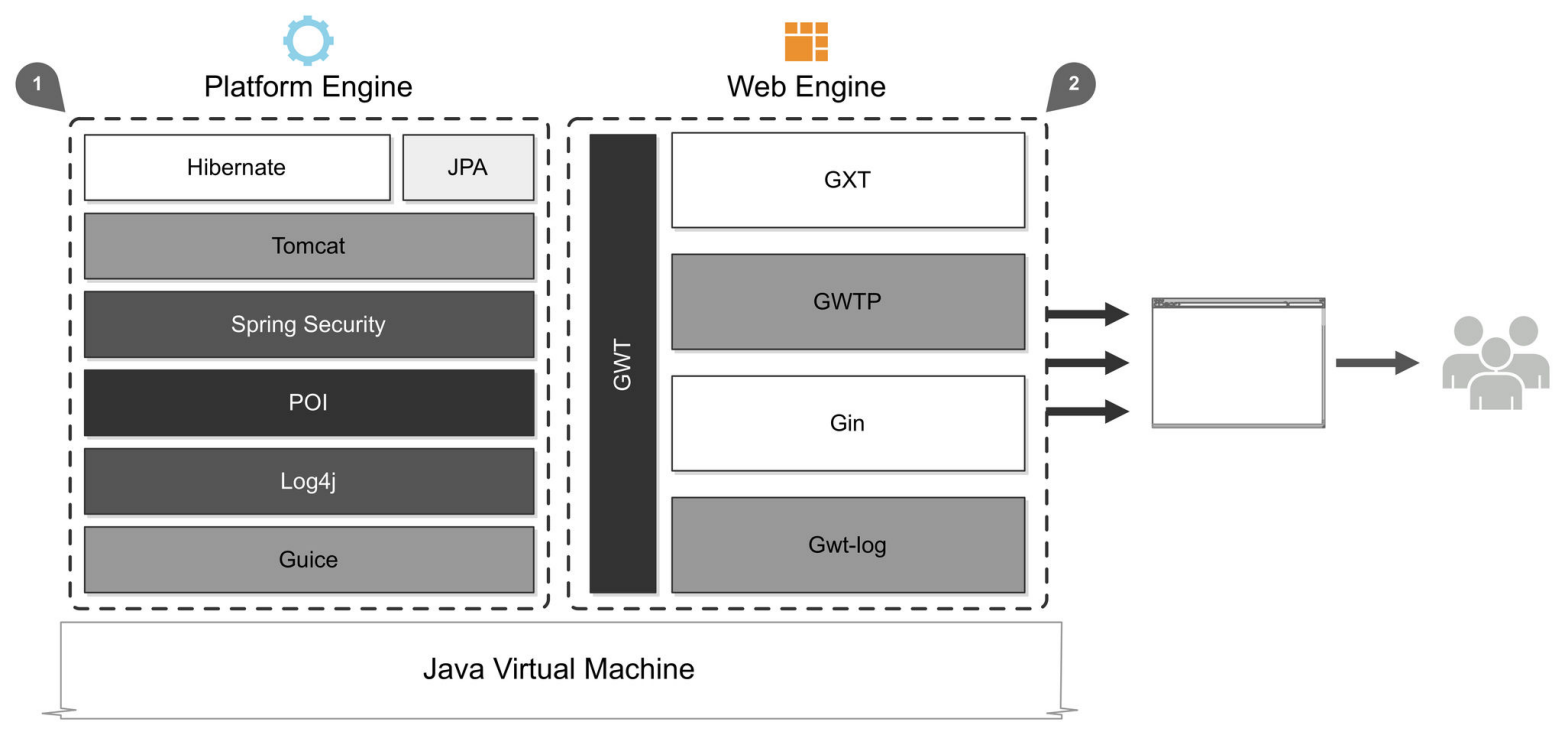
Internal platform implementation software overview. Used components are implemented in Java, leveraging the open-source nature of this solution. 1) Platform engine components include Hibernate, JPA, Spring Security, POI, Log4j, Guice and custom code to control the application and serve it as a Tomcat web application. 2) The Web engine relies on Google Web Toolkit to generate a highly responsive web workspace. Add-ons such as GXT and Gin were used to improve the user interactions’ performance and reliability.

Accessing knowledge and executing available features are key challenges behind pharmacovigilance software [43]: managed data and knowledge providers' algorithms must be accessible at all times. To accomplish this, the architecture relies on two advanced components: the platform engine and the web engine. The former is the main application controller, coordinating all the others components. The latter manages the presentation layer, providing access to a web-based workspace. The implementation of both is detailed in Figure 4.

Taking into account the accessibility and interoperability requirements, the platform engine is implemented as a Java web application. For improved data handling, Hibernate (http://www.hibernate.org/) was used as a data abstraction layer and object/relational mapper, thus reducing database coupling with the application. This shields the development from future changes in the domain model storage system and eases the use within the Java object-oriented environment.

Additional components were also used, such as Spring Security (http://static.springsource.org/spring-security/site/) for improved security features, Apache POI (http://poi.apache.org/) for enhanced data import and export, Log4j for logging purposes and Apache Maven (http://maven.apache.org/) for project dependency management, building and deployment.

The platform engine mediates the interactions within the distributed ecosystem. It controls the entire architecture and its data flows, moving the data from the knowledge base towards the service execution engine, establishing secure connections in all transactions, and regulating the provider registry system, among others. In a sense, the platform engine is an intelligent proxy, coordinating everything that happens with the distributed platform internal components.

The web application engine adopts a Model-View-Presenter pattern and is implemented with the Google Web Toolkit (GWT) framework (https://developers.google.com/web-toolkit/). GWT compiles Java code to a browser-targeted JavaScript representation, resulting in an extremely effective web application. In addition, various user interaction components were added to provide a cleaner perspective over the huge datasets and easy access to data analysis and exploration features. To improve on GWT’s user interactions library, the Ext GWT package (GXT) (http://www.sencha.com/products/gxt) was used. This extends the widgets bundled with GWT core distribution to provide a more complete set of user interaction features required by the presentation layer. The combination of GWT’s basic widgets with GXT ones was further improved with Google Gin (http://code.google.com/p/google-gin/) for dependency injection, achieving a decoupled architecture.

#### Security

Security is a primary concern for any new software, especially considering the rigorous constraints of this field, for both researchers and private pharmaceutical companies [44, 45]. In the proposed architecture, security measures are applied at three levels: interactions, data and users.

All interactions within the distributed platform go through a secure HTTP channel. Knowledge providers’ services must be deployed in a HTTPS endpoint and the use of valid certificates is enforced. However, this measure only secures the execution of workflows within the service execution engine. Hence, in addition to securing the knowledge providers, the implemented architecture also delivers secure access to the web workspace, which is served through and HTTPS channel.

At the data level, managed data are securely stored in the knowledge base using encryption measures to obfuscate the actual content. For instance, data owner details are used to “salt” the data, further bolstering its illegibility. By using a cloud-based approach, the logical storage is decoupled from the physical storage, further improving the overall security. Moreover, security and privacy features are delegated to the cloud-based controller. Likewise, partner data introduced in the system has already been carefully anonymized.

At last, on a user perspective, collaboration preferences can be tightly controlled. The distributed platform collaboration features facilitate granular data sharing, so that researchers know whom they are sharing data with and how those data are being used. Considering the sensible nature of the majority of stored data, access to the web workspace requires registration and all actions are tracked, assisting the monitoring of everything that happens within the system.

## Results

The pharmacovigilance context opens various opportunities to build new data analysis and exploration ecosystems. With collaboration from partners within the EU-ADR project it was possible to implement a prototype of the proposed distributed platform. The involvement in a large-scale European project allowed for the implementation of real-world algorithms by multiple knowledge providers. This partnership brought access to a comprehensive dataset of drugs, events and statistical data. The resulting EU-ADR Web Platform, is available online at http://bioinformatics.ua.pt/euadr/. This portal is being used within and beyond the EU-ADR project scope, generating successful research results in various areas, such as the identification of drug agents causing acute myocardial infarction [46].

### Pharmacovigilance Algorithms

The initial service-oriented architecture implementation includes four knowledge providers, each with its own pharmacovigilance algorithm and made available as a Taverna workflow using secure services. The algorithms are deployed independently in distinct physical and logical settings. The first three algorithms provide a score, between 0 and 1, for each input signal, marking whether or not there is scientific evidence behind the drug-event pair.

The first algorithm, literature analysis, adopts a semantics-based approach [47] that processes Medline annotations looking for particular MeSH terms and metadata related to the submitted drug-event pair. Using the MeSH thesaurus, matches for the subheadings “chemically induced” and “adverse effects” are searched in associated publications. The “Pharmacological Action” knowledge from MeSH thesaurus is also used to refine the search.

When no matches are found, the partial scoring for the given drug-event pair is 0 (zero). In the opposite, with 3 or more publications found, the signal is scored with 1 (one). Between 0 and 3 (exclusive) publications, the partial score will be of 0.5. Positive scores imply that scientific literature has been published on the association between the drug and the event. In these cases, the knowledge provider annotates the output with PubMed ID links of the discovered publications.

The second strategy, involves a signal filtering co-occurrence process, evaluating the relationships between drugs and side effects that might have been reported previously in Medline literature, DailyMed [48] or DrugBank [49]. Data from these resources are previously indexed, including titles and abstracts from Medline, summary product characteristics from DailyMed, and ATC codes with potential adverse events from DrugBank. The algorithm then performs a chi-square test to determine if the co-occurrence of the given drug-event pair is different than what would be expected by chance.

Similarly to the first algorithm, when interactions are found in the indexed knowledge base, the signal gets a scoring of 1 (one). The annotation section of the output will include identifiers and connections to the relevant resources (Medline, DailyMed or DrugBank).

The third algorithm, signal substantiation, generates a network based on the drug-event pair containing the interactions with proteins targeted by the drug and associated events, and with biological pathways [50]. This results in drug-target and event-target profiles that are searched for common sets of proteins, the intersecting portion of the graph. 

The output of this algorithm, a comprehensive list of proteins and pathways related to the drug-event pair, is annotated to the knowledge provider output along with the partial signal classification score.

Once the data are processed through these algorithms, the results must be combined to better assess the plausibility of a given drug-event relationship. The fourth algorithm, evidence combination, uses the scores from the other knowledge providers to arrive at a degree of belief that takes available evidence into account. The algorithm uses the Dempster–Shafer theory [51] to evaluate the initial data combined with algorithm results to reach a measurable belief level that a particular drug-event pair has a low, medium or high risk. Algorithms weight and relevance in the final measurement can be customized to better fit the research context. This final risk measurement is the most important outcome of the performed pharmacovigilance research as it summarizes the relative risk for each drug-event pair in context of available knowledge.

These algorithms have been deployed independently by EU-ADR project partners, which reinforce the proposed platform suitability to environments requiring software interoperability.

### Web Workspace

EU-ADR Web Platform’s key feature is the execution of advanced post-marketing adverse drug reaction studies. Researchers upload and investigate drug-event datasets, create targeted drug studies and work with their peers through the available collaboration features. Each researcher has its own personal workspace, where they can browse existing datasets (personal or shared); upload custom drug-event pair datasets; or create drug-specific datasets, based on the overall platform data. 

**Figure 5:**
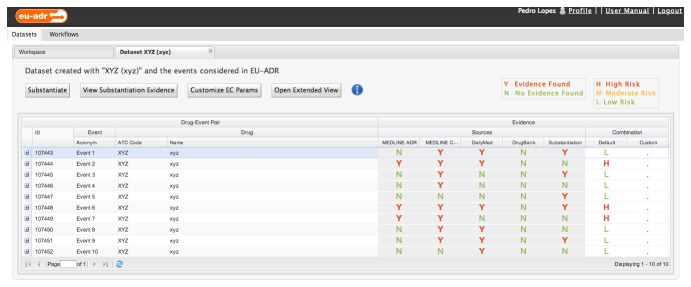
EU-ADR Web Platform workspace interface for an undisclosed drug (*XYZ*) exploration scenario containing the signal list that results from distributed knowledge provider algorithm outputs and evidence combination statistical analysis. Workflow results are labelled with Y in case sufficient evidence is found to support a potential drug-event relationship, or N otherwise. Evidence combination yields a score of H, M or L, indicating High, Moderate or Low risk respectively, of a drug-event relationship being in fact an ADR signal.

A researcher interested in studying potential adverse reactions of patients treated with a given drug, *XYZ* for the purpose of this discussion, begins its study by automatically generating a dataset focused on the targeted drug. The system then combines this drug with the 11 potential adverse events considered in EU-ADR’s context, evaluates the resulting dataset using the available knowledge providers and combines all individual pieces of evidence into an aggregate score representing the predicted risk of each drug-event relationship – Figure 5. Signals classified as moderately or highly risky should be further investigated by analysing presented evidence and following hyperlinks to biomedical literature, as well as to external drug and biological data resources.

## Conclusions

Despite the thorough research and development standards, post-market pharmacovigilance plays a key role in the assessment of existing medicines and creation of new drugs. Nevertheless, research over the last decades has focused on identifying and measuring specific adverse drug reactions in a post-marketing stage [52-54]. The holistic assessment of widespread electronic medical records empowers valuable insights over adverse drug events. Notwithstanding the value of these data *per se*, the development of new strategies to fully exploit the scientific background regarding reported events is vital.

This manuscript details the creation of such strategy, proposing a pharmacovigilance-focused distributed platform and introducing an open framework for the better exploration of the wealth of available pharmacovigilance data by all pharmacogenomics stakeholders. The EU-ADR Web Platform is a unique tool that allows researchers to exploit the wealth of data from a European cohort, combined with independent drug-event datasets. In addition to being a step forward relative to existing solutions [55], the designed strategy accurately tackles multiple challenges behind the development of state-of-the-art software within the pharmacovigilance domain: scalability, interoperability, management, reproducibility, accessibility and security.

• The plugin-based provider registry ensures that the platform is scalable. Where the standard defines the knowledge providers’ interfaces, the provider registry stores metadata regarding the available algorithms, making them available as workflows for local or remote execution.
• A new interoperability language was developed to ensure that all knowledge providers understand the data being exchanged, enabling accurate interactions within the distributed platform ecosystem.
• With knowledge providers managed through provider registry, collected data are stored in a cloud environment, streamlining the associated knowledge management tasks [56].
• This proposal enables research reproducibility through the collection of multiple datasets, which include easily reproducible analysis results. This step is further improved through the use of a cloud-based knowledge base, storing all gathered and submitted data, and ensuring availability, reliability and an eased access for all the architecture components.
• The platform’s data analysis and exploration features are accessible through a web interface, constantly available to every researcher in any kind of system or device.
• This new architecture enforces the establishment of secure communication channels amongst the platform and the knowledge providers, the security of datasets and the restricted web-based workspace.

A prototype implementation of this strategy is in place in the context of the European EU-ADR project, extending the interoperability amongst project partners. The EU-ADR Web Platform connects distributed knowledge analysis algorithms, and is available online for public use at http://bioinformatics.ua.pt/euadr/. 
